# Applications of the Crystallographic Search and Analysis System CRYSTDAT in Materials Science

**DOI:** 10.6028/jres.094.007

**Published:** 1989

**Authors:** T. Siegrist

**Affiliations:** AT&T Bell Laboratories 600 Mountain Avenue Murray Hill, NJ 07974

**Keywords:** crystallographic database, CRYSTDAT, high *T*_c_ superconductors

## Abstract

Numerical database systems have recently become available online. Their enhanced search capabilities and fast retrieval of data make them a valuable tool in research. In particular, CRYSTDAT which is a search and analysis system for NBS CRYSTAL DATA has proven to be powerful in the identification of crystalline materials. In conjunction with a single-crystal x-ray diffractometer, a qualitative as well as quantitative phase determination is easily performed. The use of CRYSTDAT will be illustrated in several examples.

## Introduction

Numerical database systems compiling inorganic crystallographic data are well established. Among the best known are the JCPDS Powder Diffraction File [1], Structure Reports (Strukturbericht) [2] and Crystal Data Determinative Tables [3]. To retrieve data from the databases in printed form, elaborate and sometimes limited and inconvenient search methods are required. In contrast, online computer systems are highly efficient. CRYSTDAT [4], which is a search and retrieval system for NBS CRYSTAL DATA [5] is accessible online and makes use of efficient search algorithms. It is therefore possible to search many parameter fields efficiently and the retrieved data can be quickly evaluated. In the following, several examples on the possible use of CRYSTDAT will be given. These examples only cover a small part of the different search routines available and use a subset of the options implemented.

## Applications

Currently, NBS CRYSTAL DATA contains unit cell, composition, formula, symmetry, and reduced cell data of approximately 115,000 inorganic and organic compounds up to 1985. Using CRYSTDAT, each of these data fields can be searched individually or they can be combined through logical operators. The examples will be restricted to inorganic oxides, illustrating possible uses of CRYSTDAT in the research on high *T*_c_ ceramic superconductors. In particular, the first example will show how the database was used in the study of the Ba-Y-Cu-O system. After logging into the account and loading the database, the system replies:

**Table:** 

ON-LINE SYSTEM	
DATE: 88-06-16	Copyright U.S. Department of Commerce, National Bureau of Standards, 1985
DATABASE: CRYSTDAT	
START TIME: 20:08:25	

The number of entries for the CRYSTDAT database = 115067

The coverage is for the years 1900–1985

### 1. Study of the Ba-Y-Cu-O System

> find class i (find inorganic compounds)
  –Set 1 created with 59951 hits
>set limits 1 (limits following searches to set 1)
  –Limit Set=1
>find ele O (find oxides)
  –Set 2 created with 31062 hits
>set limits 2
  –Limit Set = 2 (limits following searches to set 2)

#### a. Search for Ternary Ba-Cu-Oxides

>find ele Ba.and.Cu.and.3 (search for ternary barium cuprates)
  –Set 3 created with 3 hits
>show 3 (present the results of set 3)

**Table:** 

ID^1^: 803667	– 1
RC : a=5.72 b = 5.72 c = 6.46 al= 116.3 be= 116.3 ga = 90.0	
CD: sys=tetragonal spgr(CD)=I41/amd spno = 141 den=6.0(g/cc) z=4	
EM: Ba Cu2 O2	
FO : Ba Cu2 O2	
NM: Barium dicopper(i) oxide	
AC: a=5.722 c= 10.064 sprg(A)=I41/amd	
RF : Z. Naturforsch. B,27,296,1972	
ID : 708260	– 2
RC : a= 15.81 b= 15.81 c= 15.81 al= 109.5 be= 109.5 ga= 109.5	
CD: sys = cubic spgr(CD) = I432 spno = 211 den = 7.6(g/cc) z=0	
EM: BaCu O2	
FO : Ba Cu O2	
NM: Barium Copper Oxide	
AC: a= 18.26 spgr(A) = I432	
RF : Rev. Chim. Miner., 13,440,1976	
ID : 800744	– 3
RC : a= 15.82 b= 15.82 c= 15.82 al= 109.5 be= 109.5 ga= 109.5	
CD: sys = cubic spgr(CD) = Im3m spno = 229 den = 5.6(g/cc) z = 90	
EM: Ba Cu O2	
FO : Ba Cu O2	
NM: Barium dioxocuprate	
AC : a= 18.27 spgr(A) = Im3m	
RF : Z. Naturforsch. B,32,121,1977	

#### b. Search for Ternary Cu-Y-Oxides

>find Y.and.Cu.and.3
  –set 4 created with 2 hits
>show 4

**Table:** 

ID : 711441	– 1
RC : a=3.50 b= 10.80 c= 12.46 al=90.0 be = 90.0 ga = 90.0	
CD: sys = orthorhombic spgr(CD) = Pn21a spno=33 den = 5.4(g/cc) z=4	
EM: Cu2 O5 Y2	
FO : Cu2 Y2 O5	
NM: Copper Yttrium Oxide	
AC : a= 10.799 b = 3.4960 c= 12.456 spgr(A) = Pna21	
RF : 00GRNT,,,1981	
ID : 811630	– 2
RC : a = 3.52 b = 3.52 c= 11.42 al = 90.0 be=90.0 ga= 120.0	
CD: sys = hexagonal spgr(CD) = P63/mmc spno=194 den=5.0(g/cc) z = 2	
EM: Cu O2 Y	
FO : Cu Y O2	
NM: Copper (i) yttrium oxide	
AC: a =3.5206 c= 11.418 spgr(A)=P63/mmc	
RF : J. Solid State Chem.,49,232,1983	

#### c. Search for Ternary Ba-Y-Oxides

>find ele Y.and.Ba.and.3
  –Set 5 created with 4 hits
>show 5

**Table:** 

ID : 705238	– 1
RC : a=3.45 b= 10.39 c= 12.11 al=90.0 be = 90.0 ga=90.0	
CD: sys = orthorhombic spgr(CD) = Pnab spno = 60 den = 7.6(g/cc) z = 0	
EM: Ba O4 Y2	
FO : Ba Y2 O4	
NM: Barium Yttrium Oxide	
AC : a=10.388 b = 12.110 c=3.448 spgr(A) = Pnab	
RF : Mater. Res. Bull.,9,1631,1974	
ID : N108141	– 2
RC: a = 3.45 b= 10.41 c= 12.12 al=90.0 be = 90.0 ga=90.0	
CD: sys=orthorhombic spgr(CD) = Pnam spno = 62 den=5.8(g/cc) z=4	
EM: Ba O4 Y2	
FO : Ba Y2 O4	
NM: Barium yttrium oxide (1 ^~^ 2 *^~^* 4)	
AC: a= 10.415 b= 12.120 c=3.455 spgr(A) = Pnam	
RF : Z. Naturforsch. B,19,955,1964	
ID : 705239	– 3
RC : a=4.38 b=4.38 c= 11.85 al = 90.0 be = 90.0 ga=90.0	
CD: sys=tetragonal den=8.2(g/cc) z = 0	
EM: Ba2 O5 Y2	
FO : Ba2 Y2 O5	
NM: Barium Yttrium Oxide	
AC: a=4.3771 c= 11.852	
RF : Mater. Res. Bull.,9,1631,1974	
ID : 706037	– 4
RC : a=6.11 b = 6.11 c=25.17 al = 90.0 be=90.0 ga=120.0	
CD: sys=hexagonal den = 2.0(g/cc) z=0	
EM: Ba3 O9 Y4	
FO : Ba3 Y4 O9	
NM: Barium Yttrium Oxide	
AC: a=6.1102 c = 25.172	
RF : Mater. Res. Bull.,9,1631,1974	

#### d. And Finally Search for the Quaternary Ba-Y-Cu-Oxides

>find ele Ba.and.Y.and Cu.and.4
  –Set 6 created with 1 hits
>show 6

**Table:** 

ID : 809291	– 1
RC: a=5.66 b = 7.13 c= 12.18 al = 90.0 be = 90.0 ga=90.0	
CD: sys=orthorhombic spgr(CD) = Pbnm spno = 62 den = 6.2(g/cc) z=4	
EM: Ba Cu O5 Y2	
FO : Y2 Ba Cu O5	
NM: Diyttrium barium copper oxide	
AC: a=7.132 b = 12.181 c=5.658 spgr(A)=Pbnm	
RF : J. Solid State Chem.,43,73,1982	

Now we can (attempt to) construct a preliminary phase diagram of the Ba-Y-Cu-O system that serves as the starting point of the analysis of this phase space.

#### e. Identification of a Crystalline Material

In the course of studying the phase system, small black crystals were obtained. From 6 reflections measured on the CAD4 diffractometer, a unit cell of 3.495 × 6.23 × 10.795 Å was inferred. This unit cell serves as the starting point for a database search to identify the phase. In this case, the reduced cell is searched with a preset tolerance to retrieve all phases with similar lattice parameters.

> cells (*calculate the reduced cell and set up the search parameters*)
—Input Cell—
a ? > 3.495
b ? > 6.23 (*if blank, default is the previous value*)
c ? > 10.795
alpha ? > *(the default is 90* °)
beta ? > *(if blank, default is the previous value)*
gamma ? >
Lattice Type ? *(default P)* >
Tolerance ? *(default 0.10)* >

**Table:** 

> Input cell+vol.	3.50	6.23	10.80	90.00	90.00	90.00	235.05
> Lattice type	P						
>Red. Cell + Vol	3.49	6.23	10.79	90.00	90.00	90.00	235.05
> Niggli Matrix	12.215	38.813	116.532				
	0.000	0.000	0.000				
> Metric Symmetry	Orthorhombic						
> Tolerance for cell match	0.100						

Search the data base? (yes/no) > y

–Set 7 created with 0 hits

*The database was searched with the reduced cell of the lattice with edges of 3.49, 6.23, and 10.795* Å. *No matches were found. However, the unit cell determined may be too small (a subcell) because some reflections could have been missed on the diffractometer. The database is searched again, but this time, possible supercells are included in the search.*

> cells

—Input Cell—

a ? > 3.495
b ? > 6.23
c ? > 10.795
alpha ? >
beta? >
gamma ? >
Lattice Type ? *(default P)* >
Tolerance ? *(default 0.10)* >

**Table:** 

> Input cell+vol.	3.50	6.23	10.80	90.00	90.00	90.00	235.05
> Lattice type	P						
>Red. Cell+Vol.	3.49	6.23	10.79	90.00	90.00	90.00	235.05
> Niggli Matrix	12.215	38.813	116.532				
	0.000	0.000	0.000				
> Metric Symmetry	Orthorhombic						
> Tolerance for cell match	0.100						

Search the data base? (yes/no) > n

*The database is not searched with the original cell Instead, a supercell search* [*6*] *will be carried out. CRYSTDAT is instructed to generate possible supercells of* 2× *the volume of the input cell. After the supercells have been generated, the database is searched.*

Sub-/Super-cells calculated? (YES/NO)
  where sub-cell (input cell vol.) / multiplicity
  and super-cell (input cell vol.) * multiplicity > y
Sub-cell ? (yes/no) > n
Super-cell ? (yes/no) > y
Multiplicity ? (*2/3/4/5/6/7… etc*)>2

Super-cells of 2 times the volume of the input cell

**Table:** 

a	B	c	al	be	ga	vol
3.495	6.230	21.590	90.0	90.0	90.0	470
3.495	12.460	12.464	120.0	90.0	90.0	470
6.230	6.990	11.347	107.9	90.0	90.0	470
6.990	7.143	11.347	81.3	72.1	60.7	470
3.495	10.795	12.460	90.0	90.0	90.0	470
6.990	7.143	10.795	90.0	90.0	119.3	470
6.230	6.990	10.795	90.0	90.0	90.0	470

Search the data base? (yes/no) > y

**NOW SEARCHING**

*The database is now searched including all the above given supercells of multiplicity 2.*

–Set 8 created with 31 hits

In this set of retrieved entries, we find the following one:

**Table:** 

ID : 711441	– 1
RC : a=3.50 b= 10.80 c= 12.46 al = 90.0 be=90.0 ga=90.0	
CD: sys=orthorhombic spgr(CD) = Pn21a spno = 33 den = 5.4(g/cc) z=4	
EM: Cu2 O5 Y2	
FO : Cu2 Y2 O5	
NM: Copper Yttrium Oxide	
AC: a= 10.799 b=3.4960 c=12.456 spgr(A) = Pna21	
RF : 00GRNT,,,1981	

Supercell 5 from the above list of supercells matched a cell in the database of a compound with the expected elemental composition. In this way, both the correct lattice and an accurate composition of the small black crystals were determined in roughly 30 minutes.

### 2. Study of the Bi-Sr-Ca-Cu-O System

In the case of the Bi-Sr-Ca-Cu-O superconductors, the analogous searches are easily done.

#### a. Search for Ternary Bi-Cu Oxides

>find ele Bi.and.Cu.and.3
  –Set 9 created with 4 hits
>show 9

**Table:** 

ID : B034138	– 1
RC : a=5.80 b=8.48 c = 8.48 al = 90.0 be = 90.0 ga = 90.0	
CD: sys = tetragonal spgr(CD) = P4/ncc spno=130 den = 8.0(g/cc) z = 2	
EM: Bi4 Cu O7	
FO : Bi4 Cu O7	
NM: Bismuth copper oxide (4^~^1^~^7)	
AC: a = 8.481 c = 5.803 spgr(A) = P4/ncc	
RF : An. Acad. Brasil. Cienc.,38,35,1966	
ID : 804574	–2
RC : a = 5.81 b = 6.67 c = 6.67 al = 79.0 be = 64.1 ga = 64.1	
CD: sys = tetragonal spgr(CD) = I4 spno = 79 den = 8.6(g/cc) z = 4	
EM: Bi2 Cu O4	
FO : Cu Bi2 O4	
NM: Copper dibismuthate(iii)	
AC: a = 8.484c = 5.813 spgr(A) = I4	
RF : Z. Anorg. Allg. Chem.,426,1,1976	
ID : 704311	–3
RC : a = 5.81 b = 8.51 c = 8.51 al = 90.0 be = 90.0 ga=90.0	
CD: sys = tetragonal spgr(CD) = P4/ncc spno=130 den = 8.6(g/cc) z = 4	
EM: Bi2 Cu O4	
FO : Cu Bi2 O4	
NM: Copper Bismuth Oxide	
AC : a = 8.510 c = 5.814 spgr(A) = P4/ncc	
RF : C. R. Hebd. Seances Acad. Sci. Ser. C,276,l105,1973	
ID : 810281	–4
RC : a=5.81 b = 8.51 c = 8.51 al = 90.0 be = 90.0 ga = 90.0	
CD: sys = tetragonal spgr(CD) = P4/ncc spno=130 den = 8.6(g/cc) z = 4	
EM: Bi2 Cu O4	
FO : Cu Bi2 O4	
NM: Bismuth copper oxide	
AC : a=8.510 c = 5.814 spgr(A) = P4/ncc	
RF : Bull. Soc. Fr. Mineral. Cristallogr.,99,193,1976	

#### b. Search for Ternary Bi-Sr-Oxides

>find ele Bi.and.Sr.and.3
  –Set 10 created with 4 hits
>show 10

**Table:** 

ID : B028466	– 1
RC : a=3.95 b = 3.95 c = 9.64 al = 78.2 be = 78.2 ga=60.0	
CD: sys = rhombohedral den = 6.0(g/cc) z = 0	
EM: Bi38 O59 Sr2	
FO : Bi38 Sr2 O59	
NM: Bismuth strontium oxide (38 ^~^2 ^~^59)	
AC : a=3.952 c = 28.09 spgr(A) = R	
RF : J. Res. Nat. Bur. Stand. Sect. A,68,197,1964	
ID : 805401	– 2
RC : a = 3.97 b = 3.97 c=9.75 al = 78.2 be = 78.2 ga = 60.0	
CD: sys=rhombohedral spgr(CD) = R-3m spno=166 den=7.8(g/cc) z=9	
EM: Bi0.76 Ol.38 Sr0.23	
FO : Bi0.765 Sr0.235 Ol.383	
NM: Bismuth strontium oxide (.8/.2/1.1)	
AC: a=9.75 al = 23.49 spgr(A) = R-3m	
RF : J. Solid State Chem.,35,192,1980	
ID : 812013	–3
RC : a=3.97 b = 3.97 c = 9.74 al = 78.2 be = 78.2 ga = 60.0	
CD: sys=rhombohedral spgr(CD) = R-3m spno=166 den=7.8(g/cc) z = 4	
EM: Bil.72 O3 Sr0.53	
FO : Bil.72 Sr0.53 O3	
NM: Bismuth(iii) strontium(13/4) oxide	
AC: a = 3.971 c = 28.41 spgr(A) = R-3m	
RF : Solid State Ionics,3,457,1981	
ID : 709884	–4
RC : a=4.26 b = 9.60 c = 9.60 al=87.2 be = 77.2 ga = 77.2	
CD: sys = tetragonal spgr(CD) = I4/m spno = 87 den = 7.2(g/cc) z = 0	
EM: Bil. 10 O2.55 Sr0.90	
FO : Sr0.9 Bil.l O2.55	
NM: Strontium Bismuth Oxide	
AC: a=13.239 c=4.257 spgr(A) = I4/m	
RF : Rev. Chim. Miner.,15,153,1978	

#### c. Search for Bi-Cu-Sr or Bi-Cu-Ca Compounds with 4 or 5 Elements

>find ele Bi.and.Cu.and.Sr.and.5
  –Set 11 created with 0 hits
>find ele Bi.and.Cu.and.Sr.and.4
  –Set 12 created with 0 hits
>find ele Bi.and.Ca.and.Cu.and.4
  –Set 13 created with 0 hits
>find ele Bi.and.Cu.and.Ca.and.5
  –Set 14 created with 0 hits

No entries containing 4 and 5 elements were found.

#### d. Find materials with lattices related to that of a superconducting crystal

*From the x-ray study of a superconducting crystal, we obtained a cell of approximately 5.44 × 5.43 × 30.8* Å. *This cell data can be used to retrieve compounds that may be structurally related to the superconductors. For this purpose, tetragonal or orthorhombic cells are retrieved and searched for their c-axis values given by the authors.*

*Carry out several searches to generate SET 17 containing inorganic materials with Bi and O; restrict next search to this set.*

>Find sys O.or.T *(orthorhombic or tetragonal systems only)*
  –Set 18 created with 244 hits
> Set limits 18 *(limits now set to inorganic bismuth oxides belonging to the orthorhombic or tetragonal systems*)
  –Limit Set = 18
>find cc 28.to.34 *(find compounds with the author’s c-axis between 28 and 34* Å)
  –Set 19 created with 8 hits

*In this way, compounds with unit cell c-axes between 28 to 34* Å *are retrieved more efficiently than by using the “cells” command.*

>show 19

**Table:** 

ID : 711644	– 1
RC : a=3.83 b=3.83 c = 33.62 al = 90.0 be = 90.0 ga=90.0	
CD: sys = tetragonal den = 7.4(g/cc) z = 0	
EM: Bi3 F O11 Pb Ti3	
FO : Pb Bi3 Ti3 O11 F	
NM: Lead Bismuth Titanium Oxide Fluoride	
AC: a = 3.833 c = 33.62 spgr(A) = P4/mm	
RF : J. Solid State Chem.,36,349,1981	
ID : B019359	–2
RC : a = 3.84 b = 3.84 c= 16.64 al = 96.6 be = 96.6 ga = 90.0	
CD: sys = tetragonal spgr(CD) = I4/mmm spno=139 den=8.0(g/cc) z = 2	
EM: Bi4 O12 Ti3	
FO : Bi4 Ti3 O12	
NM: Bismuth titanium oxide (4^~^ 3 ^~^12)	
AC: a = 3.841 c = 32.83 spgr(A) = I4/mmm	
RF : Ark. Kemi, 1,499,1949	
ID : B024961	– 3
RC : a=3.92 b = 3.92 c= 14.71 al=97.7 be = 97.7 ga=90.0	
CD: sys = tetragonal den = 7.2(g/cc) z=2	
EM: Bi3 Br3 Ca O4	
FO : Bi3 Ca Br3 O4	
NM: Bismuth calcium bromide oxide (3^~^1 ^~^3 ^~^4)	
AC : a=3.92 c = 28.90 spgr(A) = I	
RF : Z. Anorg. Allg. Chem.,248,121,1941	
ID : B024960	–4
RC : a=3.98 b = 3.98 c= 14.66 al=97.8 be = 97.8 ga = 90.0	
CD: sys = tetragonal spgr(CD) = I4/mmm spno=139 den = 7.4(g/cc) z=2	
EM: Bi3 Br3 O4 Sr	
FO : Bi3 Sr Br3 O4	
NM: Bismuth strontium bromide oxide (3^~^1 ^~^3 ^~^4)	
AC: a=3.976 c=28.78 spgr(A) = I4/mmm	
RF : Z. Anorg. Allg. Chem.,246,115,1941	
ID : B024962	– 5
RC : a=4.04 b=4.04 c= 16.23 al = 97.2 be = 97.2 ga = 90.0	
CD : sys = tetragonal spgr(CD)=I4/mmm spno= 139 den = 7.4(g/cc) z = 2	
EM: Bi3 13 O4 Sr	
FO : Bi3 Sr I3 O4	
NM: Bismuth strontium iodide oxide (3^~^1^~^3^~^4)	
AC: a=4.043 c = 31.95 spgr(A)=I4/mmm	
RF : Z. Anorg. Allg. Chem.,250,173,1942	
ID : 709204	–6
RC : a = 5.40 b = 5.44 c=29.05 al = 90.0 be=90.0 ga = 90.0	
CD: sys=orthorhombic den=3.0(g/cc) 2=0	
EM: Bi7 Nb O21 Ti4	
FO : Bi7 Ti4 Nb O21	
NM: Bismuth Titanium Niobium Oxide	
AC : a = 5.44 b = 5.40 c=29.05 spgr(A) = P63	
RF : J. Less-Common Metals,48,319,1976	
ID : 805515	–7
RC: a = 5.41 b = 5.45 c =16.64 al = 99.4 be = 90.0 ga = 90.0	
CD: sys = orthorhombic spgr(CD) = C2ca spno = 41 den = 8.0(g/cc) z = 4	
EM: Bi4 O12 Ti3	
FO : Bi4 Ti3 O12	
NM: Tetrabismuth trititanium oxide	
AC: a = 5.448 b = 5.411 c = 32.83 spgr(A)=B2cb	
RF : Ferroelectrics,3,17,1971	
ID : N110983	– 8
RC: a = 5.41 b=5.45 c = 32.82 al = 90.0 be=90.0 ga = 90.0	
CD: sys = orthorhombic den=8.0(g/cc) 2 = 4	
EM: Bi4 O12 Ti3	
FO : Bi4 Ti3 O12	
NM: Bismuth titanium oxide (4 ^~^3^~^12)	
AC: a = 5.411 b= 5.449 c = 32.82	
RF : J. Electrochem. Soc,116,832,1969	

As it turns out, the last entry served as a starting point for the successful refinement of the Bi-Sr-Ca-Cu-O superconductor phase.

The set of commands a user has to remember is small and the commands themselves are self explanatory making CRYSTDAT an efficient tool in materials research.

## Footnotes

1 Output abbreviations are as follows: ID = compound identification no., RC = reduced cell, CD = Crystal Data, EM = empirical formula, FO=chemical formula, NM = name, AC=author’s cell, RF=journal reference.
